# Prosthetic rehabilitation of maxillary and mandibular gunshot defects with fixed basal implant-supported prostheses: A 5-year follow-up case report

**DOI:** 10.1016/j.ijscr.2020.02.025

**Published:** 2020-02-19

**Authors:** Fadia Awadalkreem, Nadia Khalifa, Abdelnasir G. Ahmad, Ahmed Mohamed Suliman, Motaz Osman

**Affiliations:** a Department of Oral Rehabilitation, Prosthodontics Division, Faculty of Dentistry, University of Khartoum, Sudan; b Chair of the Department of Preventive and Restorative Dentistry, University of Sharjah, Faculty of Dental Medicine, Sharjah, Sharjah, United Arab Emirates; c International University of Africa, Oral and Maxillofacial Surgery Department, Khartoum, Sudan; d Department of Oral and Maxillofacial Surgery, Faculty of Dentistry, University of Khartoum, Khartoum, Sudan; e Implant Department, Khartoum Teaching Dental Hospital, Federal Ministry of Heath, Khartoum, Sudan

**Keywords:** FPD, fixed partial dentures, BCS, basal cortical screw, Gunshot injury, Mandibular defect, Defect resection, Basal implant-supported prosthesis, Aesthetic results, Functional outcomes

## Abstract

**Introduction:**

Gunshot injuries to the oral maxillofacial region have serious aesthetic and functional consequences.

**Presentation of case:**

A 32-year-old male patient presented with a marginal mandibular resection owing to a gunshot wound. A clinical examination revealed destroyed crowns in teeth 12, 11, and 21; extraction in teeth 13, 22, 24, 31, 32-45; a fracture in tooth 38; and severe pain. Further, he had multiple submental scars and a completely obliterated sulcus. Consequently, a multidisciplinary team was formed who devised the following treatment plan: stage 1, root canal treatment for the maxillary anterior teeth, followed by crown construction and transitional mandibular removable partial denture construction, and stage 2, placement of immediately loaded basal implant definitive prostheses supported by 6 corticobasal screw implants. After 5 years of use, the patient presented with excellent peri-implant soft tissue health, prosthesis stability, and great improvements in aesthetics and function. The patient was highly satisfied with the treatment and restoration of his quality of life.

**Discussion:**

Accurate treatment planning is vital for managing complicated cases, including gunshot defect cases, and should involve the selection of the most suitable reconstructive technique with lower susceptible complication rates and high success rates. The described treatment eliminates the need for bone grafting, reduces treatment duration, and involves fixed prosthesis placement.

**Conclusion:**

To our knowledge, this is the first report on basal implant-based full-mouth rehabilitation in a gunshot mandibular defect patient. The fixed hybrid basal implant-supported prosthesis produced highly acceptable aesthetic and phonetic results and greatly improved the patient’s life.

## Highlights

• Oral maxillofacial gunshot wounds have serious aesthetic and functional consequences.
• Rehabilitating mandibular gunshot defects is complicated and requires a long time.
• A multispecialty team is necessary to perform challenging rehabilitation procedures.
• Fixed basal implant-supported prosthesis placement does not require bone grafting.
• This procedure produces excellent aesthetic/functional outcomes and implant stability.

## 1 Introduction

Mandibular defects commonly result from congenital deformities, tumour resection, or trauma. Maxillofacial gunshot injuries may not result in life-threatening trauma, but in most cases, are associated with aesthetic configuration-, masticatory dysfunction-, speech- or deglutition-related issues, affecting patient quality of life. Additionally, patients may present with difficulties in saliva control and compromised tongue movement [[1], [2], [3]]. Rehabilitating such patients can be frustrating and challenging [[1], [2], [3]].

The use of surgical and/or prosthetic reconstructive techniques to rehabilitate mandibular defects has been governed by the quality and quantity of the remaining soft and hard tissue, the loss of mandibular continuity, number and distribution of the remaining teeth, adjacent vital structures [4], vestibular obliteration, donor site feasibility and morbidity for bone graft procedures, costs, availability of an expert maxillofacial surgeon, and patient age, medical condition, and preferences [2,[4], [5], [6]].

Futran et al.’s [7] treatment protocol for avulsion wounds, including gunshots, consists of three phases. The first phase involves an urgent assessment of the “ABC” life support parameters, wound cleaning, foreign body extraction, infectious tissue removal and sequestration, and the stabilization of fractured bones accompanied by primary wound closure and formulating a definitive treatment plan. Phase 2 is the definitive reconstructive phase. Phase 3 involves the final cosmetic and functional refinement of the reconstructive prosthesis [7].

A one-stage treatment protocol involving wound site debridement and a composite tissue flap as an immediate reconstructive technique has also been described [1].

The main reconstruction goals are to maintain hard and soft tissue integrity and to replace the missing structures, for which, fractures can be fixed using open or close reduction techniques [5].

With dental implant-supported prostheses, the restoration of patient aesthetics, mastication, and phonation has extensively improved. Contrarily, implants require bone anchorage onto the remaining bony structures following resection or via bone grafting [2,[8], [9], [10]]. Considering the grafting procedure- and implant-related complications, a need arises for an adjunctive treatment option with lesser complications.

Basal implants are commonly used in cases involving reduced bony support as they can be deeply anchored into the basal bone via their horizontal plates [[11], [12], [13], [14], [15]]. Usually implants connected with a metal framework for better force distribution increase the feasibility of immediate loading and permit the use of both ceramic and acrylic denture base materials. A great advantage of this implant system is that all the forces are transmitted through the vertical shaft deep into the strongest basal bone [[11], [12], [13], [14], [15]]. These features qualify this system for use in extensive mandibular defect patients. In this report, we have described the prosthetic rehabilitation of a mandibular gunshot defect patient using implant-supported fixed partial dentures (FPD) supported by 6 basal cortical screw implants and a maxillary fixed bridge.

This work has been reported in line with the SCARE criteria [16]. Ethical approval to conduct the work was obtained from the authors’ institute.

## 2 Presentation of case

**Fig. 1:**
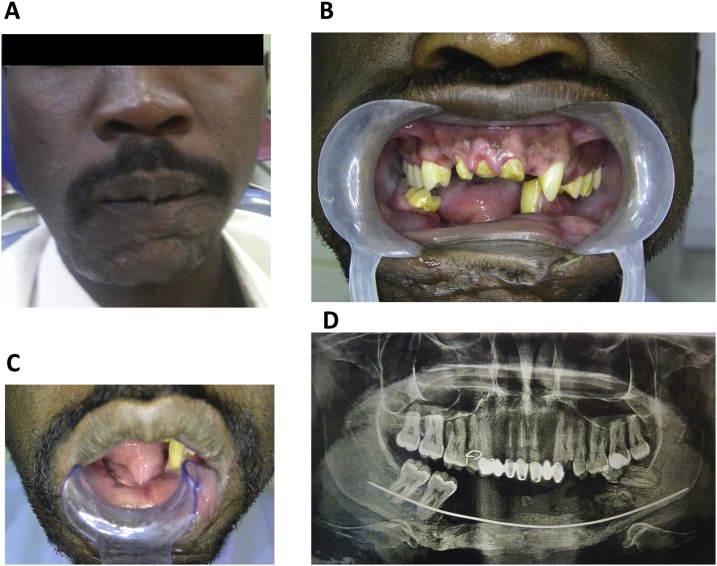
Patient’s clinical presentation. A. The patient’s photograph presents his frontal view at the time of presentation with a slight facial deformity and multiple scars on the submental region. B. The intraoral view presenting severely destroyed crowns in teeth 12, 11, and 21; missing teeth 13, 22, 45, 44, 43, 42, 41, 31, and 32; and a fractured crown in tooth 38. C. The intraoral view of the patient with an obliterated sulcus. D. The panoramic radiograph shows the  presence of a supporting wire and a fracture in crown 38.

A 32-year-old man with an anterior mandibular defect and severely destroyed maxillary anterior teeth resulting from a gunshot wound was referred to the Department of Prosthodontics of the authors’ institution (Fig. 1a). A clinical examination revealed complete intraoral soft tissue healing; severely destroyed crowns in teeth 12, 11, and 21; the extraction of teeth 13, 22, 24, 31-32, and 41-45; a fracture in tooth 38; and severe pain (Fig. 1b). He had multiple submental scars extraorally and a completely obliterated sulcus intraorally (Fig. 1c). A radiographic evaluation using the digital panoramic view (Planmeca Pro max, Finland) revealed a supporting wire (Fig. 1d). A multidisciplinary team was formed, and the treatment plan involved the following: stage 1, root canal treatment for the maxillary anterior teeth followed by crown construction and transitional mandibular removable partial denture construction for functional (mastication, phonation) and cosmetic purposes and stage 2, definitive prosthesis placement based on the remaining mandibular bone quantity and on the patient’s preference to not undergo another major bone grafting procedure, his finances, and treatment expectations. We decided to use a mandibular-fixed prosthesis supported by 6 basal cortical screw (BCS®, Dr. Ihde Dental AG, Switzerland) implants and scheduled the patient for a follow-up. The treatment plan was discussed with him, and his approval was obtained for the treatment and publication of this report.

### 2.1 Treatment

**Fig. 2:**
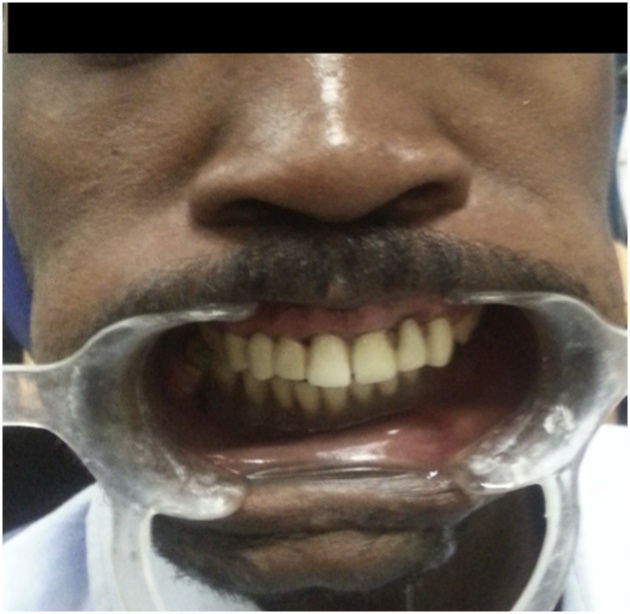
The intraoral view depicting the insertion of the transitional mandibular acrylic partial dentures and fixed maxillary bridge for the teeth 13, 12, 11, 21, and 22.

A transitional acrylic mandibular denture was constructed following the standard technique, the occlusal plane level and amount of lip support were adjusted based on aesthetic and phonetic considerations. Based on transitional denture appointments, root canal treatment was performed for the maxillary anterior teeth and bilaterally for the first premolar, followed by crown preparation after transitional denture insertion as a guide for occlusal clearance. Temporal porcelain-fused-to-metal crowns were cemented, and the final occlusal adjustment was performed. Subsequently, the patient was schedule for implant treatment (Fig. 2).

**Fig. 3:**
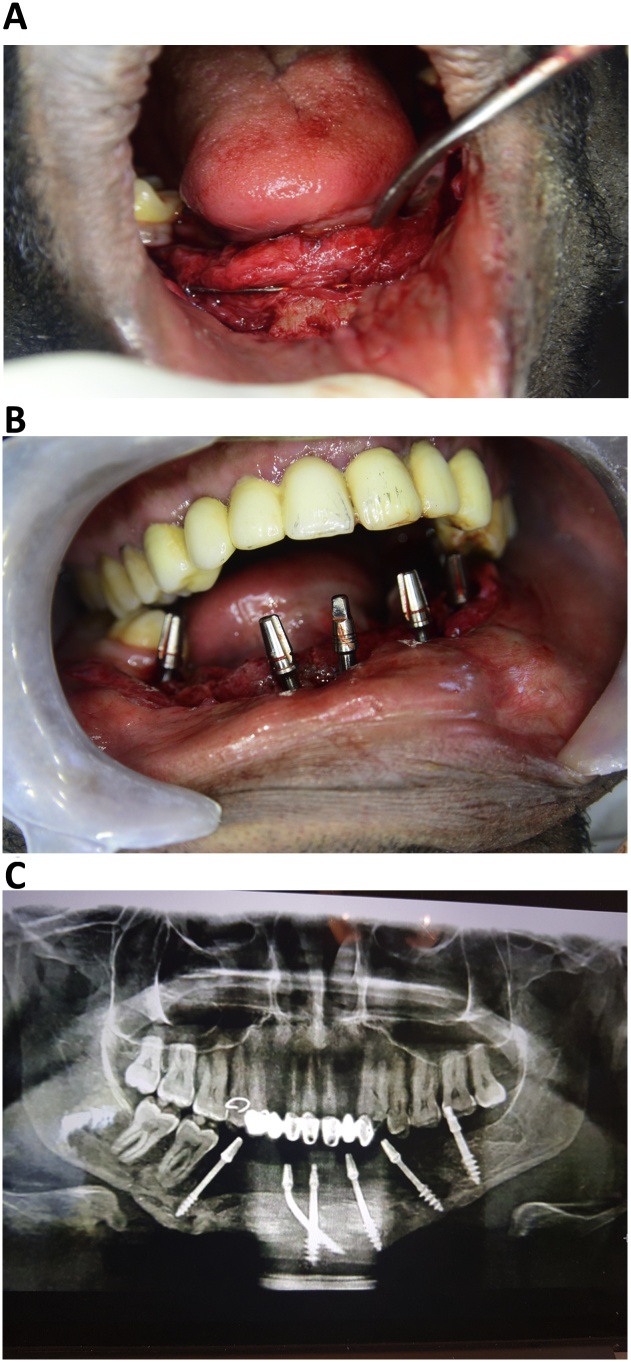
Intraoral rehabilitation of a 32-year-old man with a gunshot defect using immediately loaded basal implant-supported fixed prostheses. A. Elevation of a periosteal flap, removal of the supporting wire, and implant insertion. B. Implant distribution and flap suturing. C. Post-operative panoramic radiograph.

After 3 months, he returned for implant treatment and was asked to rinse his mouth with betadine 10% for 1 min. Local anaesthesia was induced 2% lidocaine with epinephrine 1:100000, a flap was raised, and the stabilizing wire Kitchener wire was removed Fig. 3a). Simultaneously, implant osteotomy was performed and six BSC® implants with appropriate lengths and diameters were inserted in the areas, 31, 33, 35, 37, 41, and 45. (Fig. 3b) The isoelastic implants [11,12] were bent using a bending tool supplied by the manufacturer to ensure better prosthetic alignment, and the flap was sutured. The antibiotics, amoxicillin 1 g and metronidazole 500 mg, and the analgesic, 50 mg diclofenac potassium (Rapidus), were prescribed. A digital panoramic view image was captured postoperatively (Fig. 3c).

### 2.2 Fixed implant-supported prostheses

**Fig. 4:**
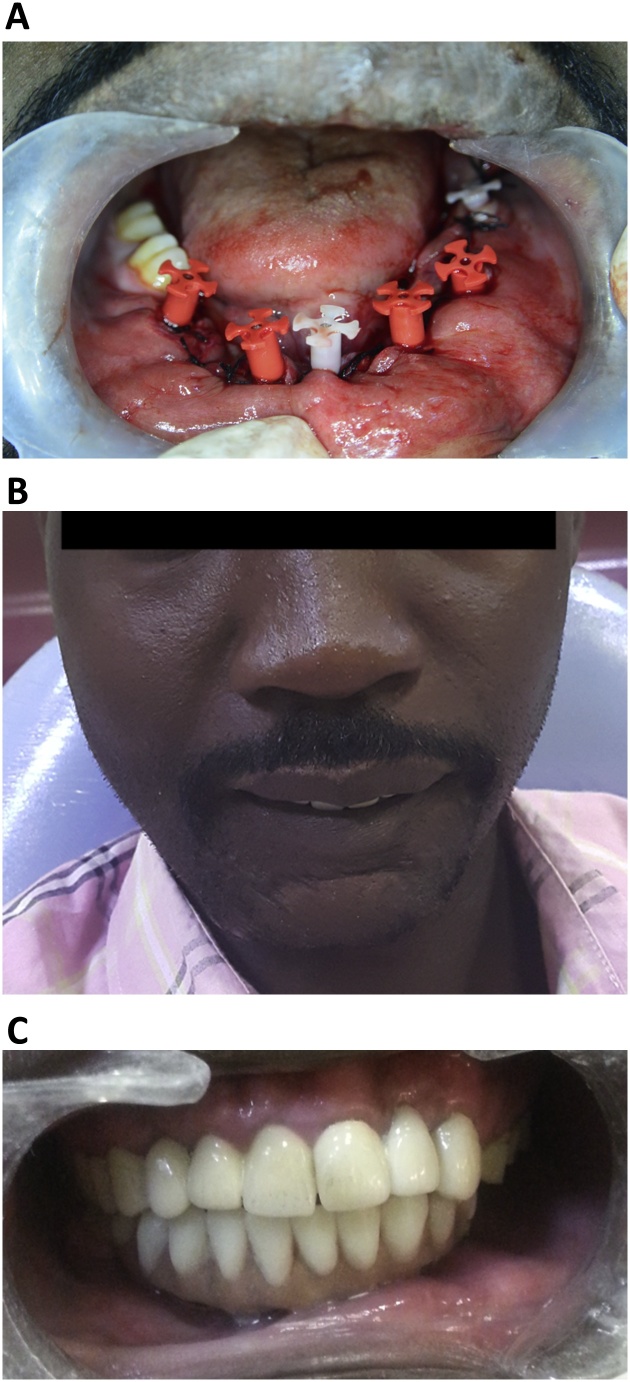
The prosthetic rehabilitation of the patient. A. The intraoral view depicting the impression copping tied over the abutments’ head. B. The frontal view depicting the final maxillary fixed bridge and final mandibular basal implant-supported prostheses. C. The intraoral view depicting the labial and lingual extensions of the mandibular implant-supported prostheses.

Impression copings were secured and the final impression was acquired using monophase (VPS; Ivoclar Vivadent AG). A day later, a metal framework was constructed to splint the implants together. The lower prosthesis comprised of acrylic resin teeth and veneer material to compensate the severe tissue loss. The prosthetic labial and lingual extensions were not extended to the full depth of the sulcus, thereby providing a hygienic space, preventing food entrapment, and ensuring the salivary washing action. On the third day, the finished prosthesis was inserted and cemented using Fuji cement (GC Corporation, Tokyo, Japan) (Fig. 4b). The patient was educated on routine cleaning and oral hygiene maintenance, including the use of a very soft small-headed interdental toothbrush to clean the prosthetic mucosa through the hygienic space (Fig. 4c).

**Fig. 5:**
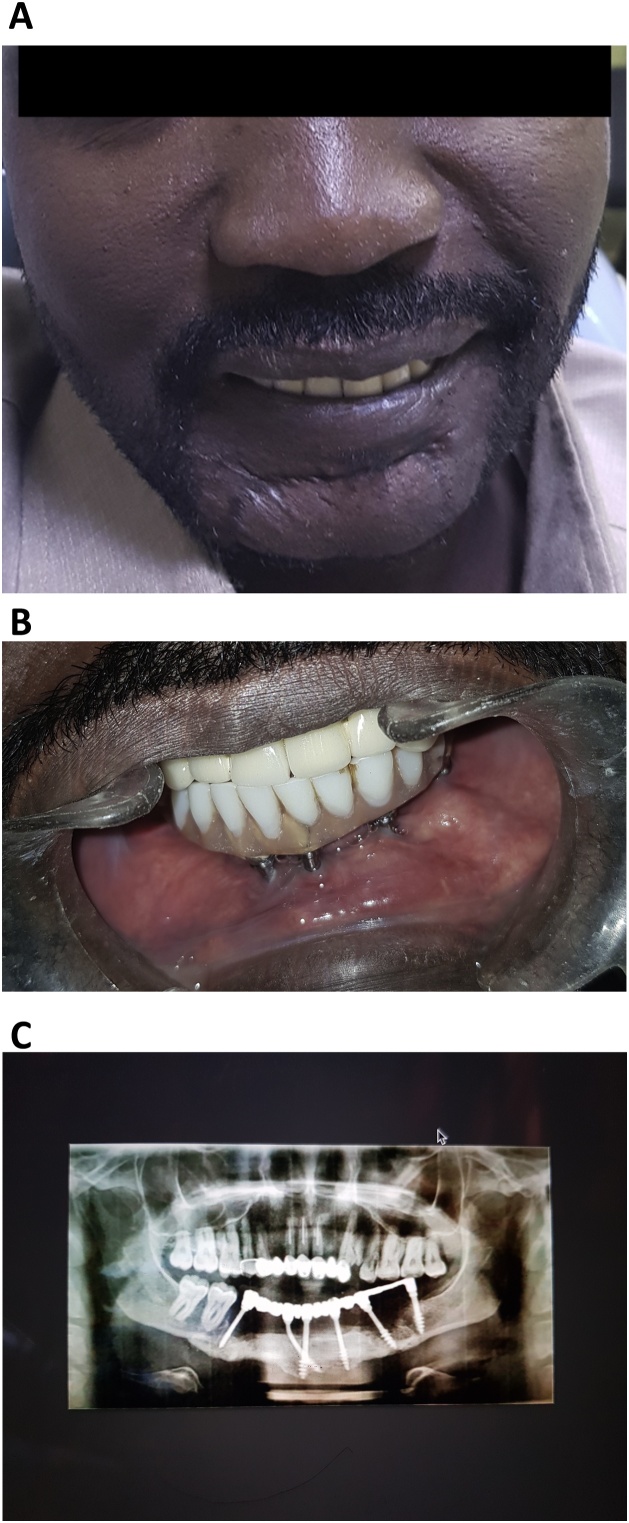
Follow-up images of the immediately loaded basal implant-supported fixed prostheses fabricated for a 32-year-old man with a gunshot defect. A. The frontal view of the patient after 5 years of follow-up. B. The intraoral view of the patient depicting his clinical presentation exhibiting optimal peri-implant oral health. C. A panoramic radiograph showing the maxillary and mandibular prostheses after 5 years of function.

After a week, the patient was followed-up, revealing no problems, and he was scheduled for periodic follow-ups. After 5 years, the patient exhibited excellent peri-implant soft tissue health, good prosthesis stability with a slightly chipped canine suprastructure which was dealt with, submental scar reduction, and great aesthetic and functional improvements, which restored his quality of life making him highly satisfied with the treatment (Fig. 5a–c).

## 3 Discussion

Rehabilitating a gunshot-related mandibular resection patient is a multi-step, challenging treatment procedure that requires a long time [3]. A multidisciplinary team is mandatory to ensure successful results as this trauma type commonly causes multifunctional issues involving many dental specialties [1].

Several treatment options have been considered for gunshot-related mandibular resection patients including the use of implant-supported prostheses, which can be hindered by many factors according to the previous literature [[1], [2], [3],^6^]. Nowadays, using basal implant-supported prostheses has several advantages, including eliminating the need for bone grafting and its risk factors. Further, supported prostheses can be fixed immediately, which is usually the first request of majority of patients, especially the youngest patients.

Accurate treatment planning is vital for implant treatment success in advanced cases [3] and should involve selecting the most suitable reconstructive technique with low complication and high success rates. In this case, the patient requested a treatment option involving minimal surgical intervention, high success rates, and the restoration of his aesthetics, mastication, and phonetics to their pre-trauma state. Therefore, the treatment of choice was basal implant-supported prosthesis placement [[11], [12], [13], [14], [15]].

Commonly, in mandibular defect patients, the severe ridge irregularities and inadequate soft tissue support can adversely affect implant-supported prosthetic stability and retention. Additionally, the abnormal lateral forces increase the prosthetic dislodgement force, resulting in loosening with long-term use. Consequently, using fixed hybrid prostheses was considered the best prosthetic treatment option for our patient owing to the easier handling, quick treatment duration, and metal framework that ensures better force distribution and decreases the force per implant [4]. Further, the acrylic denture base (Hybrid design) compensates for the severe soft and hard tissue loss and provides sufficient lip support, thereby yielding highly acceptable aesthetic and phonetic results. The main biological and mechanical problems commonly associated with fixed implant-supported prostheses are increased plaque accumulation, gingival hyperplasia, and peri-implantitis [1,4,[17], [18], [19], [20]]. Interestingly, these complications do not occur with BCS® implants as they have smooth surfaces, preventing plaque microorganism adherence [[11], [12], [13], [14], [15]]. Further, their small penetrating tips ensure quick peri-implant soft tissue healing and healthy peri-implant mucosa [[11], [12], [13], [14]]. Additionally, using prostheses that produce a hygienic space under the prosthesis flanges ensures the washing action of saliva and prevents remnant food and plaque accumulation. Furthermore, using acrylic resin greatly improved the patient’s frontal and lateral profiles, thereby improving his satisfaction and quality of life.

## 4 Conclusion

This report describes the clinical evaluation of a 32-year-old man with a mandibular gunshot defect and his prosthetic rehabilitation using fixed hybrid basal implant-supported prostheses with an acrylic denture base covering a metallic framework. This treatment produced effective aesthetic and functional results and greatly improved patient quality of life.

## Funding

This research did not receive any specific grant from funding agencies in the public, commercial, or not-for-profit sectors.

## Ethical approval

The research was registered at the research centre of the Khartoum Dental Teaching Hospital, Federal Ministry of Health, Khartoum, Sudan, after the approval of the research ethical committee of Khartoum Dental Teaching Hospital.

## Consent

The approval of the patient was obtained for the treatment and publication of this report.

## Author contribution

Awadalkreem F contributed to the conceptualization, treating the patient, writing, editing, finalization and submission of the case.

Khalifa N contributed to the conceptualization, validation, and supervision of the case.

Ahmad A contributed to the conceptualization, validation, treating the patient, and supervision of the case.

Suliman AM was contributed to the conceptualization validation, and supervision of the case.

Osman M contributed to the conceptualization, treating the patient, editing, and finalization of the manuscript.

## Registration of research studies

The research was registered at the research centre of the Khartoum Dental Teaching Hospital, Federal Ministry of Health, Khartoum, Sudan, after the approval of the research ethical committee of Khartoum Dental Teaching Hospital.

## Guarantor

Fadia Awadalkreem.

## Provenance and peer review

Not commissioned, externally peer-reviewed.

## Declaration of Competing Interest

The authors declare no conflicts of interest in connection with this research and manuscript.
